# Variations in ADH and ALDH in Southwest California Indians

**Published:** 2007

**Authors:** Cindy L. Ehlers

**Keywords:** Alcohol use disorder, alcohol and other drug (AOD)-related (AODR) mortality, AOD use, abuse, and dependence, alcoholism, East Indians, Native American, Southwest California Indians, genetic factors, genetic polymorphisms, protective factors, alcohol flush reaction, ethanol metabolism, alcohol dehydrogenase (ADH), aldehyde dehydrogenase (ALDH), acetaldehyde, ALDH1, ALDH2, ALDH2*2, ALDH1A1*2, ADH1B, ADH1B*3

## Abstract

Native Americans as a group have the highest rates of alcohol-related deaths of all ethnicities in the United States; however, it remains unclear how and why a greater proportion of individuals in some Native American communities develop alcohol-related problems and alcohol use disorders (AUDs). One potential factor that can influence responses to alcohol are variations in alcohol-metabolizing enzymes. Researchers have analyzed the frequencies of variants in the alcohol-metabolizing enzymes alcohol dehydrogenase (ADH) and aldehyde dehydrogenase (ALDH) in some Native American populations. So far the studies have yielded no evidence that an ALDH2 variant, which has shown protective effects in other populations, is found in either American Indians or Alaska Natives. A variant of the ALDH1 enzyme that is encoded by the ALDH1A1*2 allele, however, was found in a small proportion of a group of Southwest California Indians and had a protective effect against alcoholism in that population. Furthermore, a variant of the *ADH1B* enzyme that is encoded by the ADH1B*3 allele was found in a similar proportion of Southwest California Indians and also was associated with a protective effect. However, these findings do not explain the high prevalence of alcoholism in the tribes investigated.

Native Americans (i.e., American Indians and Alaska Natives) historically have experienced numerous alcohol-related problems since the drug’s introduction into their culture by European settlers. When investigating alcohol use and related problems among Native Americans, however, it is important to recognize the wide diversity among this population’s subgroups. Although Native American people comprise less than 1 percent of the U.S. population, there are more than 300 Federally recognized tribes distributed throughout the country. Consistent with this cultural and geographic diversity, policies regarding alcohol use and the prevalence of drinking differ among tribal nations. Some tribes have disallowed alcohol use on their reservations, others are geographically isolated from sources of alcohol, and still others have no formal policy.

Although tribes differ with regard to alcohol use, Native Americans as a group have the highest rates of alcohol-related deaths of all ethnicities in the United States. Indian Health Service reports of age-adjusted death rates attributed to alcohol are more than five times higher than those for the general U.S. population (Shalala et al. 1999). Moreover, although alcohol consumption has taken a greater toll on Native American men than on women, alcohol-related death rates in Native American women still are 3 to 10 times higher, depending on age, than in women in the general population. Despite the devastating impact that alcohol has had on some tribes, however, it remains unclear how and why a greater proportion of individuals in some Native American communities develop alcohol-related problems and alcohol use disorders (AUDs).

Over the years, several popular theories and myths have arisen concerning alcohol use and its consequences in Native American communities. One of these is the “firewater” myth—a common stereotype suggesting that “Indians can’t hold their liquor” because their bodies metabolize alcohol differently. Despite the perpetuation of this myth, few studies have tested this hypothesis experimentally. This article explores to what extent the presence of certain variants of alcohol-metabolizing enzymes may, or may not, account for the high rates of alcohol use and AUDs in a select group of Indians residing in southwestern California (Mission Indians), for whom high rates of alcohol dependence have been reported (i.e., up to 72 percent for men and 53 percent for women) (Ehlers et al. 2004*a*,*b*; Wall et al. 2003) and who have been studied intensively.

## Alcohol-Metabolizing Enzymes in Native Americans

As described in other articles in this issue, genetically influenced metabolic factors have been implicated in the etiology of alcoholism not only in Native Americans but also in other ethnic groups. The two main enzymes involved in alcohol metabolism are alcohol dehydrogenase (ADH) and aldehyde dehydrogenase (ALDH). ADH breaks down alcohol to the toxic metabolite acetaldehyde, which is then broken down further by ALDH into the less toxic acetate. There are two main types of ALDH:

- ALDH1, which is found in the fluid filling the cells (i.e., the cytosol), is produced in both the central nervous system and other tissues; in addition to participating in acetaldehyde metabolism, this enzyme is involved in the synthesis of retinoic acid, a precursor of vitamin A.
- ALDH2 is found in the small cell structures that are involved in the cell’s energy production (i.e., mitochondria); this type of ALDH is responsible for the bulk of acetaldehyde breakdown in the body.

For both ADH and ALDH, several genetically determined variants (i.e., isoforms) exist that differ in their level of activity (i.e., whether they break down alcohol or acetaldehyde faster or more slowly). (For more information on these variants and their characteristics, see the accompanying article by Edenberg in this issue.) People carrying different ADH and ALDH isoforms metabolize alcohol at different rates. For example, a person carrying a less active ADH isoform metabolizes alcohol at a slower rate than a person carrying a more active ADH isoform. The ADH and ALDH isoforms also determine the acetaldehyde levels that may build up in the body after alcohol consumption. Both a more active ADH isoform and a less active ALDH isoform can lead to acetaldehyde accumulation and enhance the adverse effects associated with higher acetaldehyde concentrations. The most severe of these adverse effects is the “flushing syndrome,” which occurs after alcohol consumption in individuals with ALDH2 mutations. It includes facial flushing, nausea, rapid heart beat (i.e., tachycardia), and other unpleasant effects.

ADH and ALDH isoforms arise from natural variations (i.e., polymorphisms) in the structure of the genes that code for these enzymes. The frequency of these polymorphisms differs among ethnic groups and has been explored in Native Americans. The first of these studies was conducted by Bennion and Li (1976), who measured the rates of alcohol metabolism and enzyme patterns in a small sample of American Indians and Whites and found no racial differences.

### ALDH2 Polymorphisms in Native Americans

One of the best understood polymorphisms of alcohol-metabolizing enzymes is associated with the ALDH2 enzyme. One ALDH2 isoform, known as ALDH2*2, which is found in approximately 40 percent of people of Far East Asian descent but rarely in Caucasians, is partially inactive because of a specific mutation in the gene encoding this enzyme. In people carrying the ALDH2*2 enzyme, alcohol consumption results in acetaldehyde accumulation, which in turn leads to a facial flushing reaction, several other physiological reactions, and an overall increased sensitivity to alcohol. As a result of these adverse reactions, carriers of ALDH2*2 exhibit particularly low rates of alcohol use and alcoholism (Wall et al. 1992). (For more information on ALDH polymorphisms in Asians, see the article by Eng et al. in this issue.)

Of the studies investigating alcohol metabolism in Native Americans, several specifically examined the frequency of the ALDH2*2 isoform in members of some tribes of Native Americans. Because some anthropologists have suggested that North American Indians arrived in the New World via the Bering Strait from Asia, some researchers further proposed that Alaska Natives might be more likely than continental American Indians to carry this enzyme variant. However, neither studies in Alaska Natives (Segal 1999) nor in any other groups of Native Americans (Mulligan et al. 2003; Novoradovsky et al. 1995; Oota et al. 2004) have supported this hypothesis so far or found the ALDH2*2 variant.

### ALDH1 Polymorphisms in Native Americans

Several isoforms of the ALDH1 enzyme exist, one of which is encoded by the gene *ALDH1A1*.^1^ Spence and colleagues (2003), who studied people with a variety of ethnic backgrounds, recently identified polymorphisms of the *ALDH1A1* gene resulting in three gene variants (i.e., alleles) named ALDH1A1*1, ALDH1A1*2, and ALDH1A1*3. Of these, the ALDH1A1*2 allele was found among the Asian, Caucasian, Jewish, and African-American individuals tested, albeit at low frequencies. The ALDH1A1*3 allele, in contrast, was found only in African Americans.

Researchers also have examined the prevalence of the ALDH1A1*2 and ALDH1A1*3 polymorphisms in Southwest California Indians. Ehlers and colleagues (2004*c*) found that approximately 6 percent of the people in this population displayed the ALDH1A1*2 allele.^2^ This finding is similar to what has been reported in other ethnic groups (Spence et al. 2003). Most importantly, the study found that Mission Indians carrying the ALDH1A1*2 allele were significantly less likely to be alcoholic or to be regular smokers than were people without this allele (Ehlers et al. 2004*c*). This finding suggests that ALDH1A1*2 protects against developing alcoholism and regular smoking in Southwest California Indians. Additionally, study participants carrying the ALDH1A1*2 allele reported lower levels of drinking when they first started drinking regularly and, when asked about the maximum number of drinks they had ever consumed over a 24-hour period, reported consuming only half as many drinks as participants carrying the ALDH1A1*1 allele. The exact mechanism whereby the ALDH1A1*2 allele influences drinking and smoking behavior is not clear. However, these data suggest that the protective association between ALDH1A1*2 and alcohol dependence is mediated in part through lower levels of alcohol consumption.

These findings in Southwest California Indians also are consistent with the idea that the *ALDH1A1* gene may influence alcohol consumption and smoking in a manner similar to what has been reported for the less active ALDH2*2 variant. Thus, it is possible that alcohol consumption in people carrying the ALDH1A1*2 allele could lead to acetaldehyde accumulation, which in turn may produce an altered response to alcohol and ultimately result in lower levels of drinking and less regular tobacco usage. In addition, study participants who carried an ALDH1A1*2 allele reported significantly less positive expectations of alcohol, which may support the idea that these people have an altered response to alcohol. To test this hypothesis, however, further studies need to be conducted that directly assess people’s responses to alcohol as a function of the *ALDH1A1* variants they carry. Additional studies also are necessary to determine whether any protective influences of the ALDH1A1*2 allele act independently on tobacco and alcohol usage.

### ADH Polymorphisms in Native Americans

As with the ALDH enzymes, researchers have identified several forms of ADH (labeled ADH1–ADH7). Furthermore, polymorphisms exist for several of the genes encoding ADH enzymes, the best known of which occur in the genes encoding ADH1. There are three types of *ADH1: ADH1A*, *ADH1B*, and *ADH1C*. For the gene encoding ADH1B, three polymorphisms have been identified and labeled ADH1B*1, ADH1B*2, and ADH1B*3. The ADH1B*2 allele is highly prevalent among Asians but occurs infrequently in most Caucasians, except for people of Jewish and perhaps Hispanic descent. A combined analysis of several studies (i.e., a meta-analysis) indicated that people carrying ADH1B*2 alleles are about one-third as likely to be alcoholic compared with people without this allele, suggesting that the allele has a protective effect (Whitfield 1997). The ADH1B*3 polymorphism overall is less common but is prevalent in African Americans, in whom it has been associated with a lack of family history of alcoholism and with altered expectations of the effects of alcohol, suggesting a protective effect for this allele as well (Ehlers et al. 2001, 2003). Similarly, the ADH1B*3 allele has been associated with protection against alcohol dependence in Afro-Trinidadians (Ehlers et al. 2007).

Researchers also have investigated whether ADH polymorphisms are present in Native Americans, specifically in the select population of Southwest California Indians. These analyses found that approximately 6 percent of the Mission Indians studied carried the ADH1B*3 polymorphism (Wall et al. 2003). As mentioned above, this polymorphism was previously identified in people of African origin; however, none of the Mission Indian participants reported any African heritage.

Among the Mission Indians, those carrying an ADH1B*3 allele were about one-third less likely to have a lifetime diagnosis of alcohol dependence than were people without this allele. In addition, for ADH1B*3 carriers the maximum number of drinks ever consumed in a 24-hour period was only about half of that reported by people without that allele (Wall et al. 2003). These observations suggest a protective effect of the ADH1B*3 allele. It has been hypothesized that the ADH enzyme encoded by the ADH1B*3 allele reduces the risk for alcoholism by causing alcohol to be metabolized more rapidly compared with ADH enzymes encoded by ADH1B*1. This could theoretically lead to a more rapid acetaldehyde production, which in turn would result in increased alcohol sensitivity and lower levels of alcohol consumption.

## Conclusions

Two main conclusions can be drawn from the studies of ADH and ALDH isoforms and their encoding genes in Southwest California Indians:

- Neither Mission Indians nor any other North American Indians have the *ALDH2* gene polymorphism seen in Asians that causes facial flushing following alcohol consumption and protects people from developing alcoholism.
- Only a small number of Mission Indians carry variants of the ALDH1 and ADH enzymes. The enzyme variants that have been identified, however, reduce drinking behavior and enhance protection from the development of alcoholism.

Thus, the studies reviewed here highlight the utility of evaluating protective factors against the development of alcoholism in populations with a high prevalence of AUDs. However, these studies do not explain the high prevalence of alcoholism in the tribes investigated.

## Footnotes

1 By convention, the names of genes encoding various proteins are written in italics, whereas the names of the proteins are written in normal font.

2 This corresponds to an allele frequency of 0.03. Every person carries two copies (i.e., alleles) of a given gene (e.g., *ALDH1A1*), one inherited from the mother and one inherited from the father, that may be identical or have different polymorphisms. Therefore, an allele of which one copy is found in 6 percent of people tested accounts for 3 percent of all alleles present in those people, corresponding to an allele frequency of 0.03.
